# Association of hemoglobin A1C with circulating metabolites in Dutch with European, African Surinamese and Ghanaian background

**DOI:** 10.1038/s41387-019-0082-0

**Published:** 2019-04-30

**Authors:** Xiang Zhang, Inge C. L. van den Munckhof, Joost H. W. Rutten, Mihai G. Netea, Albert K. Groen, Aeilko H. Zwinderman

**Affiliations:** 1 Department of Experimental Vascular Medicine, Amsterdam University Medical Center, University of Amsterdam, Amsterdam, The Netherlands; 2 Department of Internal Medicine, Radboud university medical center, Nijmegen, The Netherlands; 3 Radboud Center for Infectious Diseases, Radboud university medical Center, Nijmegen, The Netherlands; 4 Department for Genomics & Immunoregulation, Life and Medical Sciences Institute (LIMES), University of Bonn, Bonn, Germany; 5 Department of Pediatrics, University Medical Center Groningen, University of Groningen, Groningen, The Netherlands; 6 Clinical Epidemiology, Biostatistics, and Bioinformatics, Amsterdam University Medical Center, University of Amsterdam, Amsterdam, The Netherlands

**Keywords:** Pre-diabetes, Type 2 diabetes

## Abstract

**Background:**

The prevalence of type 2 diabetes mellitus (T2DM) varies significantly across ethnic groups. A better understanding of the mechanisms underlying the variation in different ethnic groups may help to elucidate the pathophysiology of T2DM. The present work aims to generate a hypothesis regarding “why do subjects with African background have excess burden of T2DM?”.

**Methods:**

In the current study, we performed metabolite profiling of plasma samples derived from 773 subjects of three ethnic groups (Dutch with European, Ghanaian and African Surinamese background). We performed Bayesian lognormal regression analyses to assess associations between HbA1c and circulating metabolites.

**Results:**

Here we show that subjects with African Surinamese and Ghanaian background had similar associations of HbA1c with circulating amino acids and triglyceride-rich lipoproteins as subjects with European background. In contrast, subjects with Ghanaian and African Surinamese background had different associations of HbA1c with acetoacetate, small LDL particle and small HDL particle concentrations, compared to the subjects with European background.

**Conclusions:**

On the basis of the observations, we hypothesize that the excess burden of T2DM in subjects with African background may be due to impaired cholesterol efflux capacity or abnormal cholesterol uptake.

## Introduction

The prevalence of type 2 diabetes mellitus (T2DM) increased rapidly worldwide during the past decades, and is strongly associated with the developing obesity pandemic^1^. Apart from the common risk factors that prevail in all populations, ethnic background is a risk factor as well^2^. In the Netherlands, subjects with a migration background showed a considerable higher incidence of T2DM compared to subjects with European background^3,4^. Among men, individuals with European, Ghanaian and African Surinamese background had T2DM prevalence of 5.0, 14.9 and 10.4%, respectively^5^. Among women, the prevalence is 2.3% (European), 11.1% (Ghanaian) and 11.5% (African Surinamese)^5^. Interestingly, the differences in T2DM prevalence across ethnic groups could not be explained by genetic variants or dietary pattern alone^6,7^. In contrast, other studies showed that the ethnic differences in T2DM prevalence were accompanied by differences in plasma amino acids and lipids. In line with this, compared to Europeans, the serum concentrations of isoleucine, phenylalanine, tyrosine and alanine were significantly higher in South Asians^8^. In another study^9^, individuals with Surinamese background were identified to have lower sphingolipids, but higher unsaturated acylcarnitines and higher amino acid levels, than Europeans.

Metabolite profiling, or metabolomics, has been widely applied to identify new biomarkers for T2DM^10,11^, predict T2DM risk^12,13^ and improve our understanding of pathophysiologic mechanisms^14,15^. The most frequently observed metabolic abnormalities in insulin resistant and T2DM subjects include elevated circulating branched amino acids (BCAAs) and aromatic amino acids (AAAs)^16–18^. A hypothesized mechanism linking elevated BCAAs to T2DM is that disturbed BCAA metabolism leads to accumulation of BCAA metabolites (e.g. 2-Aminoadipic acid), resulting in pancreatic *β* cell dysfunction^19,20^. High triglyceride and low HDL cholesterol is another frequently observed metabolic abnormality in insulin resistance and T2DM subjects^21–25^. Often dyslipidemias are viewed as consequences rather than cause of T2DM^21–23^. However, cholesterol homeostasis plays an important role in regulating pancreatic *β* cell function^26–28^. Cholesterol is taken up by pancreatic *β* cells via the LDL receptor and exported back to plasma via the ATP-binding cassette transporter A1 (ABCA1)^29^. Accumulation of cholesterol in pancreatic *β* cells leads to impairment of glucose tolerance and defective insulin secretion^26,28,29^.

The present work aimed to generate a hypothesis regarding “why do subjects with African background have excess burden of T2DM?”. As an initial step, we investigated whether the relationship between circulating metabolites and glucose tolerance varies depending on ethnicity. Regarding circulating metabolites, we focused on amino acids, ketone bodies and lipoproteins, similar to what has been reported in other studies on glycemia^17,21,30,31^. In this study, we used the hemoglobin A1C (HbA1c) level as the surrogate of glucose tolerance, since HbA1c is an index of chronic glycemia and a predictor of T2DM^32,33^. Regarding ethnicity, we focused on Dutch with European, Ghanaian and African Surinamese background and described associations between HbA1c with amino acids, ketone bodies and lipoproteins in all three ethnic groups. The results of the association analyses fuel our hypothesis regarding “why do subjects with African background have an excess burden of T2DM?”.

## Materials and Methods

### Study population

The study population was composed of three ethnic groups in the Dutch population. In particular, 217 African Surinamese and 255 Ghanaian were from the HELIUS (HEalthy Life In an Urban Setting) study^34,35^, and 301 European Dutch were from the 300-Obesity cohort^36^ from the Human Functional Genomics Project^37^.

HELIUS is a multiethnic prospective cohort study in Amsterdam, the Netherlands. Participants of HELIUS study (18–70 years old) were randomly sampled and stratified by ethnic origin through the municipal registry of Amsterdam between 2011 and 2015. A total of 25 000 participants were included at baseline. In this study, 252 subjects (99 with Ghanaian background and 153 with African Surinamese background) had diabetes. The other 220 (156 with Ghanaian background and 64 with African Surinamese background) had prediabetes and were randomly sampled from the corresponding ethnic groups.

All participants enrolled in the 300-Obesity cohort study had a BMI above 27 kg/m^2^. The exclusion criteria include a recent cardiovascular event (myocardial infarction, transient ischemic attack, stroke in the past 6 months), a history of bariatric surgery or bowel resection, inflammatory bowel disease, renal dysfunction or increased bleeding tendency, and using oral or subcutaneous anticoagulant therapy or thrombocyte aggregation inhibitors (other than acetylsalicylic acid and carbasalate calcium).

In both the HELIUS and 300-Obesity cohort study, participants were considered to have diabetes if: 1. Fasting glucose level was >7 mmol/L. 2. if a participant was using glucose-lowering medication. 3. if a participant self-reported to have been diagnosed with diabetes by a health care professional.

The criterion for prediabetes was fasting glucose >5 mmol/l or HbA1c above 5.7%.

Both studies complied with all relevant ethical regulations and in accordance with the Declaration of Helsinki (6th, 7th revisions), and all participants provided written informed consent. HELIUS and the 300-Obesity studies were approved by the Ethics Committee in Academic Medical Center (AMC) Medical and Radboud university medical center.

### HbA1c measurement

Whole blood samples were used to determine the concentration of HbA1c using HPLC technology (TOSOH, Tokyo, Japan).

### Metabolite profiling

Fasting plasma samples were collected in the clinic and stored at −80 °C. Quantification of 8 amino acids, 2 ketone bodies and 14 lipoproteins was performed by using a high-throughput NMR metabolomics platform (Nightingale, Austria)^38^. The following 14 lipoprotein subclasses were quantified: extremely large (average particle diameter >75 nm), very large (average particle diameter 64.0 nm), large (53.6 nm), medium (44.5 nm), small (36.8 nm) and very small VLDL (31.3 nm); intermediate-density lipoprotein (IDL; 28.6 nm); three LDL subclasses, i.e. large (25.5 nm), medium (23.0 nm) and small LDL (18.7 nm); and four HDL subclasses, i.e. very large (14.3 nm), large (12.1 nm), medium (10.9 nm) and small HDL (8.7 nm).

### Statistical analysis

Because this study contains three ethnic groups from two different cohort studies with different time of sampling and measurement, we cannot directly compare metabolite variables between ethnic groups. As an alternative, we performed association analyses between HbA1c and circulating metabolites within each ethnic group. The outcome variable (*y*) was concentration of a metabolite, such as amino acids, ketone bodies, or lipoprotein particles. The predictor variable (*x*) was the HbA1c concentration.

To assess the strength of associations between HbA1c and metabolites, we ran lognormal regression because the outcome variables are positive continuous data with skewed distributions. To study the dependency of ethnicity on the relationship between HbA1c and metabolic variables, we introduced ethnicity-specific intercepts and slopes into the model. We also adjusted for covariates including gender, age, diabetic status and BMI (body mass index). We centered and scaled HbA1c, age and BMI, so that one unit HbA1c means 10 mmol/mol, one unit age means 10 (years), and one unit BMI means 5 kg/m^2^. Due to missing observations in both outcome and predictor variables, we applied a Bayesian lognormal regression to handle the missing data. There are two types of missing values, which are as follows: (1) when the concentration of a metabolite is below the limit of detection, or (2) when values were rejected by the automatic sample and measurement quality control procedure in the Nightingale pipeline. All the missing observations were assumed missing at random and treated as parameters. Values were randomly drawn from a normal distribution with ethnicity-dependent mean and standard deviation. Based on a previous study^39^ the mean value and standard deviation of HbA1c is about 40 mmol/mol and 5 mmol/mol, therefore, we used exponential (0.025) and *exponential* (0.2) as prior distributions. In the same study, the mean value and standard deviation of BMI is about 27 kg/m^2^ and 5 kg/m^2^, therefore, we used *exponential* (0.04) and *exponential* (0.2) as prior distributions. Regarding the missing values that were below the limit of detection, the imputed values were constrained between zero and the minimal observed value. For the other parameters, we used regularizing prior distributions and fitted the model by running Hamiltonian Markov Chain Monte Carlo in the program Stan (version 2.18.0)^40^. We ran four Markov chains with 2000 iterations in each chain. Results were presented with the posterior mean of lognormal regression coefficient with 95% credible interval (CI). The lognormal regression coefficient can be interpreted as the proportional increase of the metabolite concentration as a result of a change of 10 mmol/mol HbA1c.

## Result

### Participant characteristics

This study included in total 773 subjects from three ethnic groups in the Dutch population. Specifically, the study population consisted of 301 European Dutch, 255 Dutch with Ghanaian background, and 217 Dutch with African Surinamese background (Table 1). Dutch with European background were older than the other two ethnic groups. There were relatively more male participants in the Dutch with European background, and in the Dutch with African Surinamese background, there were relatively more female participants. In addition, 12.6% of the participants with European background were diabetic, whereas the proportion of diabetes in subjects with African Surinamese and Ghanaian were 70.5 and 38.8%, respectively. To control for these possible confounding factors, all the results shown below were after adjusting for gender, age, BMI and diabetic status. All association statistics were provided in the supplementary Table S1, Table S2, Table S3 and Table S4.

**Table 1:** Characteristics of 773 participants with different ethnic backgrounds

	European (*N* = 301)	Ghanaian (*N* = 255)	African Surinamese (*N* = 217)	*P* value
Female (%)	44.5	52.5	65.9	<0.0001
Age (years)	67.1 ± 5.4	51.2 ± 8.3	55.2 ± 7.2	<0.0001
BMI	30.7 ± 3.5	29.6 ± 4.4	31.2 ± 5.8	<0.0001
Waist circumference (cm)	107.0 ± 9.8	98.5 ± 10.6	103 ± 13.4	<0.0001
HbA1c (mmol/mol)	41.7 ± 7.9	56.1 ± 19.7	46.6 ± 15.4	<0.0001
Diabetes (%)	38 (12.6)	99 (38.8)	153 (70.5)	<0.0001

Kruskal–Wallis test was used to calculate *P* values for age, BMI, waist circumference and HbA1c

*χ*^2^ test was used to calculate *P* values for gender and diabetes

### Association of HbA1c with circulating amino acids

Since circulating amino acids are robust markers of T2DM, we first evaluated their associations with HbA1c in Dutch with European, African Surinamese and Ghanaian background.

**Fig. 1:**
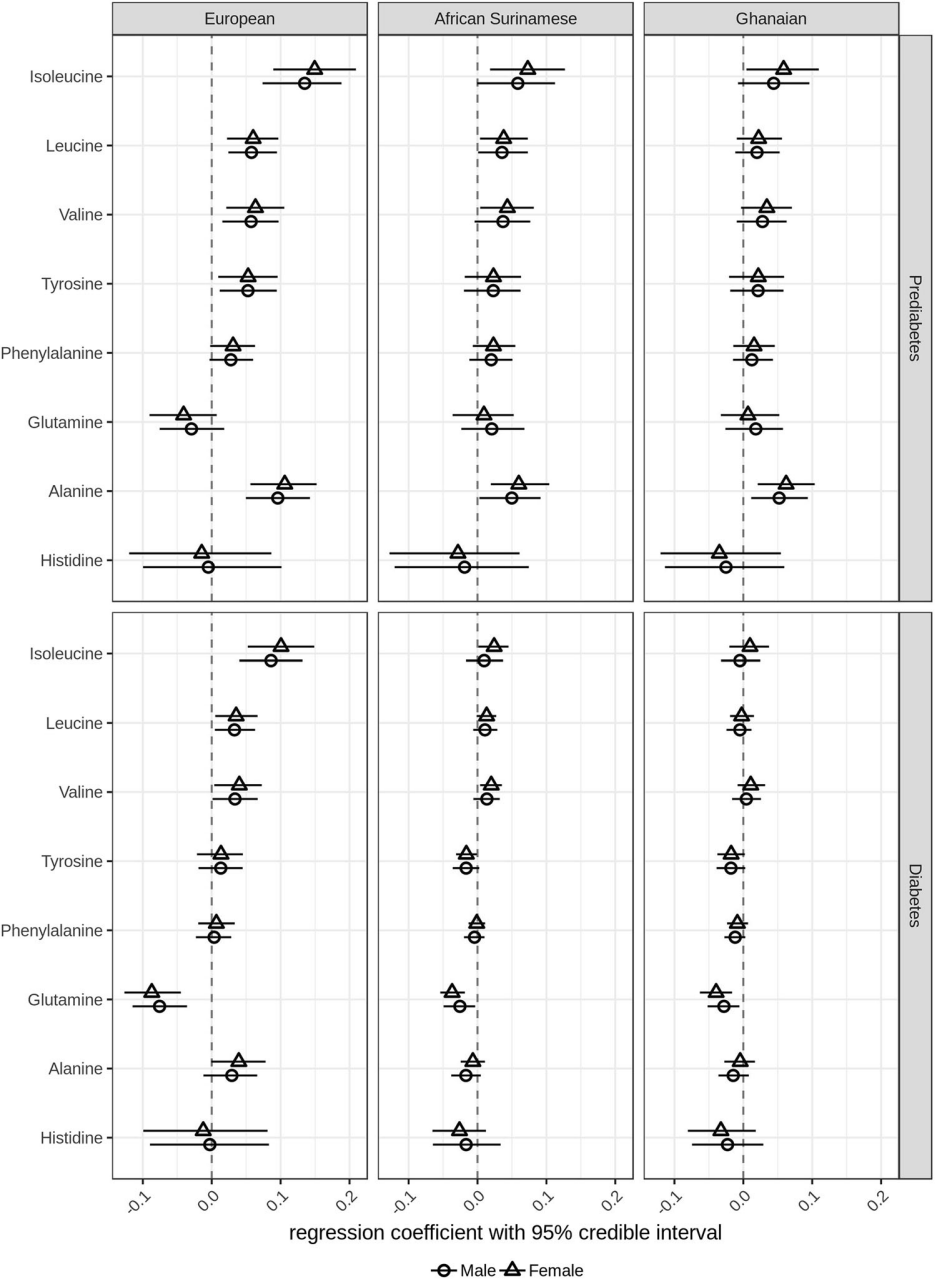
**Regression parameter estimates between plasma amino acids and HbA1c in subjects with European, Ghanaian and African Surinamese background.** Circles (female) or triangles (male) and horizontal lines represent the posterior means of the regression coefficient between plasma amino acids and HbA1c and 95% credible intervals

We observed that in prediabetic subjects with European background, increasing HbA1c concentrations were associated with increasing concentrations of circulating isoleucine (regression coefficient in males 0.14 with 95% credible interval [0.07–0.19], in females 0.15 [0.09–0.21]), leucine (males 0.06 [0.02–0.09], females 0.06 [0.02–0.09]), valine (males 0.06 [0.02–0.10], females 0.06 [0.02–0.11]), tyrosine (males 0.05 [0.01–0.10], females 0.05 [0.01–0.10]), and alanine (males 0.10 [0.05–0.14], females 0.11 [0.06–0.15]) (Fig. 1). Compared to prediabetic subjects with European background, smaller associations between HbA1c and circulating isoleucine (males 0.09 [0.04–0.13], females 0.10 [0.05–0.15]), leucine (males 0.03 [0.005–0.06], females 0.03 [0.005–0.07]), and valine (males 0.03 [0.001–0.07], females 0.04 [0.004–0.07]) were observed in diabetic subjects with European background (Fig. 1).

Similar to prediabetic subjects with European background, we observed that increasing concentrations of HbA1c were associated with increasing concentrations of isoleucine (0.07 [0.02–0.13]), leucine (0.03 [0.004–0.07]), valine (0.04 [0.004–0.08]) and alanine (0.06 [0.02–0.10]) in prediabetic females with African Surinamese background, as well as in prediabetic females with Ghanaian background (isoleucine 0.06 [0.005–0.11] and alanine 0.06 [0.02–0.10]). In diabetic females with African Surinamese background, we observed that isoleucine (0.02 [0.001–0.05]) and valine (0.02 [0.004–0.04]) remained their associations with HbA1c. Most interestingly, we observed that increasing concentrations of HbA1c were associated with decreasing concentrations of tyrosine in diabetic females with African Surinamese background (−0.02 [−0.03 to −0.001]) (Fig. 1).

### Association of HbA1c with circulating ketone bodies

**Fig. 2:**
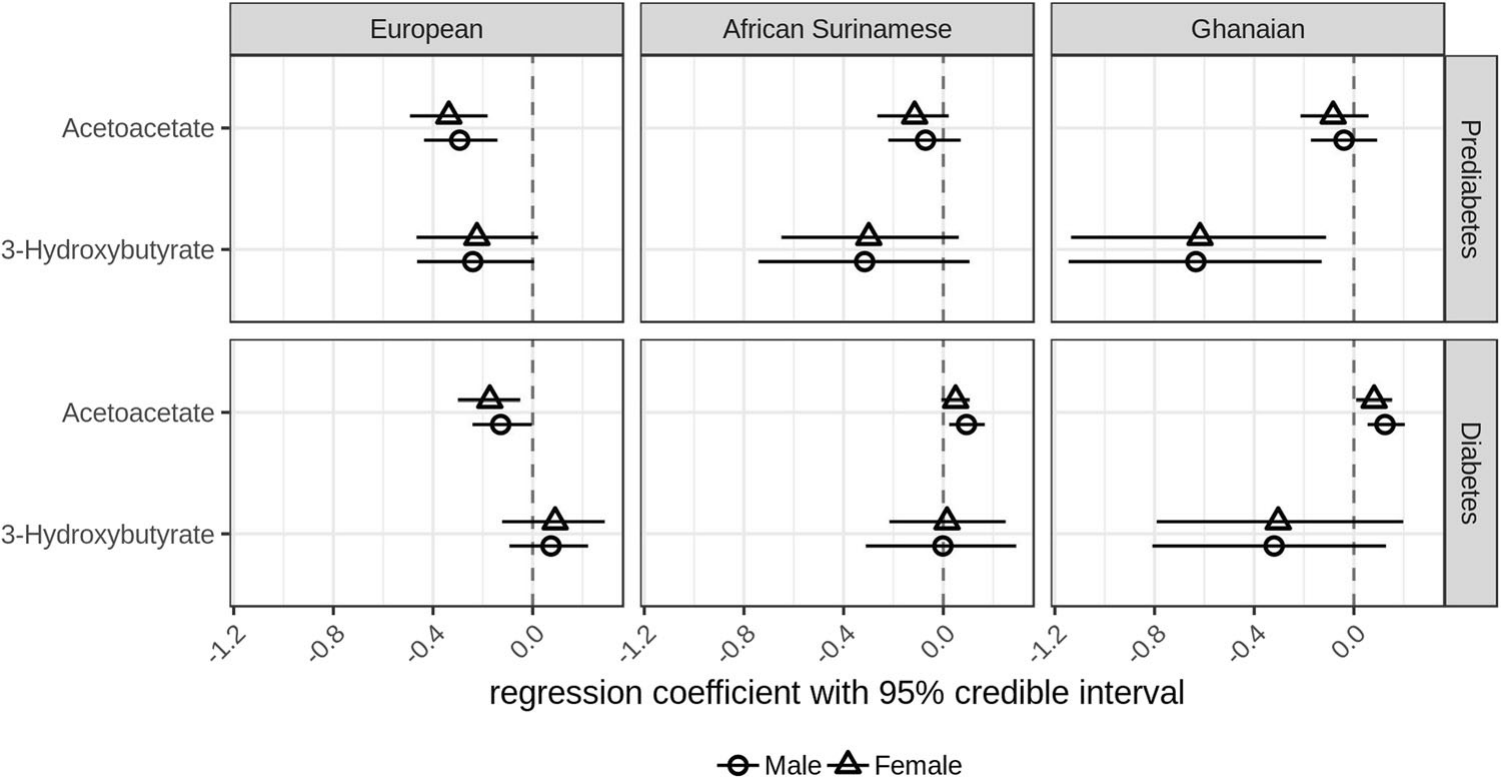
**Regression parameter estimates between plasma ketone bodies and HbA1c in subjects with European, Ghanaian and African Surinamese background.** Circles (female) or triangles (male) and horizontal lines represent the posterior means of the regression coefficient between plasma ketone bodies and HbA1c and 95% credible intervals

After circulating amino acids, ketone bodies were the second group of metabolites that we analyzed. We observed that increasing concentrations of HbA1c were associated with decreasing concentrations of acetoacetate in prediabetic subjects with European background (males −0.29 [−0.44 to −0.14], females −0.34 [−0.49 to −0.18]), but not in people with African background. Meanwhile, we observed that increasing concentrations of HbA1c were associated with decreasing concentrations of 3-hydroxybutyrate in prediabetic subjects with Ghanaian background (males −0.63 [−1.14 to −0.129], females −0.62 [−1.14 to −0.112]), but not in people with European or African Surinamese background (Fig. 2).

Among diabetic subjects, we observed that increasing concentrations of HbA1c were associated with decreasing concentrations of acetoacetate in those with European background (males −0.13 [−0.24 to −0.004], females −0.17 [−0.30 to −0.05]), but were associated with increasing concentrations of acetoacetate in African Surinamese men (0.09 [0.02–0.17]) as well as subjects with Ghanaian background (males 0.13 [0.05–0.20], females 0.08 [0.01–0.154]) (Fig. 2).

### Association of HbA1c with circulating lipoproteins

Fourteen lipoproteins (4 apolipoprotein A1 and 10 apolipoprotein B containing lipoproteins) were the third group of metabolites that we analyzed for their relation with HbA1c.

#### Apolipoprotein A1 containing lipoproteins

**Fig. 3:**
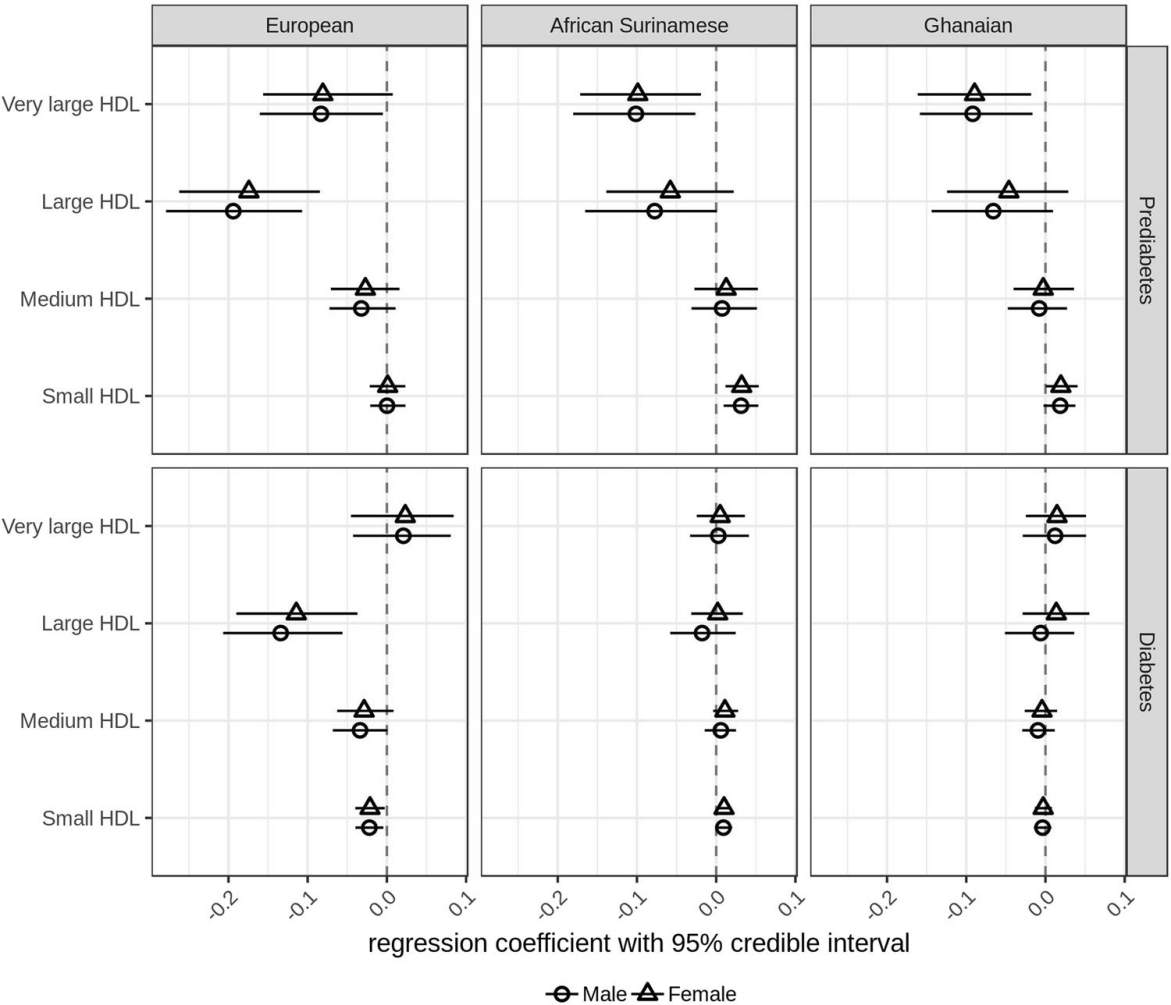
**Regression parameter estimates between plasma HDL particle concentration and HbA1c in subjects with European, Ghanaian and African Surinamese background**. Circles (female) or triangles (male) and horizontal lines represent the posterior means of the regression coefficient between plasma HDL particle concentration and HbA1c and 95% credible intervals

Four apolipoprotein A1 containing lipoproteins (very large, large, medium and small HDL particles) were measured in the Nightingale metabolomics platform. Here we observed that increasing concentrations of HbA1c were associated with decreasing concentrations of large HDL particles in prediabetic subjects with European background (males −0.20 [−0.28 to −0.11], females −0.17 [−0.26 to −0.08]), but not in prediabetic subjects with African Surinamese and Ghanaian background. In contrast to absence of associations in prediabetic subjects with European background, we noticed that increasing concentrations of HbA1c were associated with increasing concentrations of small HDL particles in prediabetic subjects with African Surinamese background (males 0.03 [0.01–0.05], females 0.03 [0.01–0.05]) as well as in prediabetic females with Ghanaian background (0.02 [0.0003–0.04]) (Fig. 3).

Similar to prediabetic subjects, we observed that increasing concentrations of HbA1c were associated with decreasing concentrations of large HDL particles (males −0.13 [−0.21 to −0.06], females −0.11 [−0.19 to −0.04]) only in diabetic subjects with European background. Furthermore, we observed that increasing concentrations of HbA1c were associated with decreasing concentrations of small HDL particles in diabetic subjects with European background (males −0.02 [−0.04 to −0.004], females −0.02 [−0.04 to −0.003]), but were associated with increasing concentrations of small HDL particles in diabetic females with African Surinamese background (0.01 [0.001–0.02]) (Fig. 3).

#### Apolipoprotein B containing lipoproteins

##### Triglyceride-rich lipoproteins

**Fig. 4:**
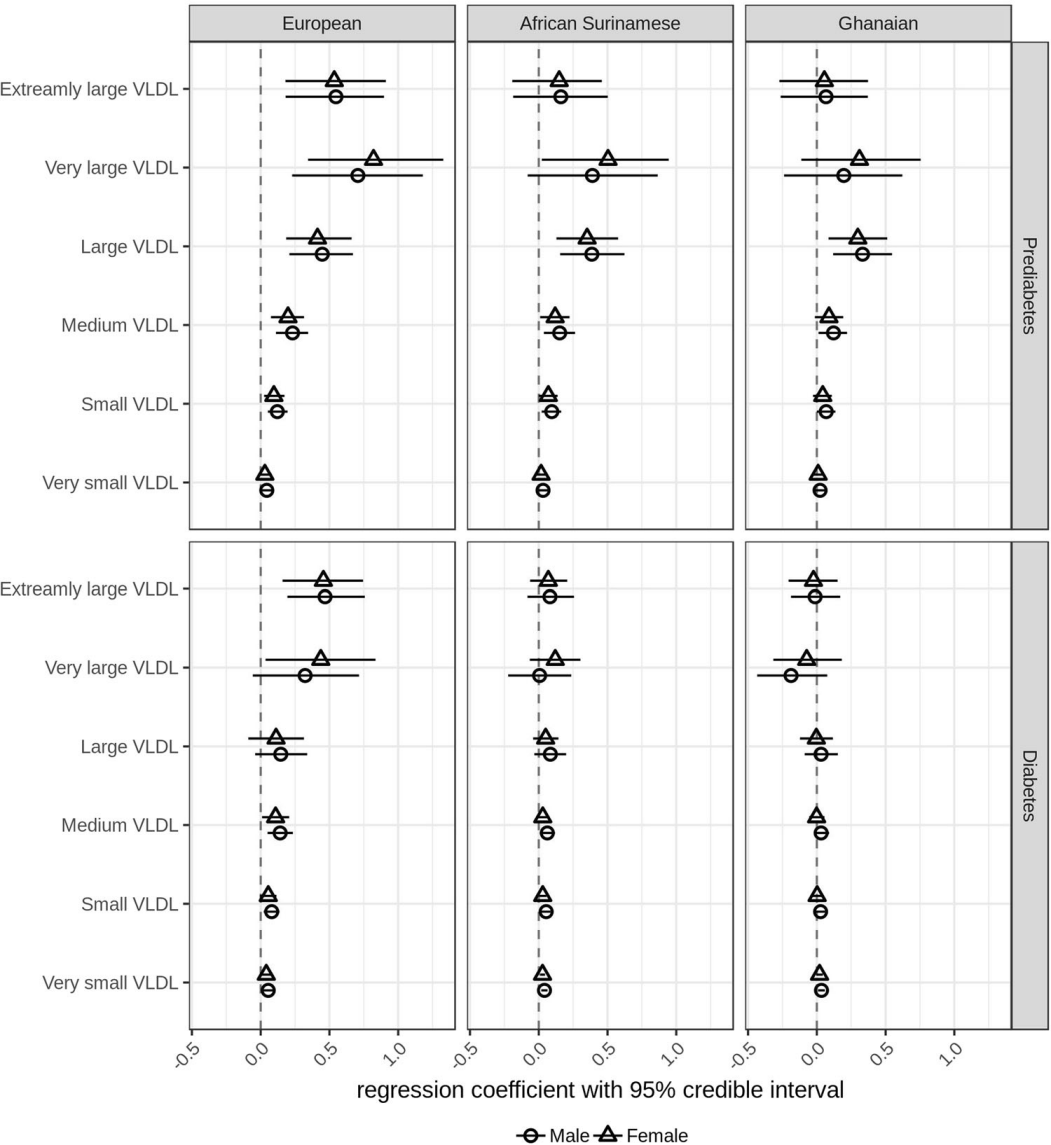
**Regression parameter estimates between plasma VLDL particle concentration and HbA1c in subjects with European, Ghanaian and African Surinamese background**. Circles (female) or triangles (male) and horizontal lines represent the posterior means of the regression coefficient between plasma VLDL particle concentration and HbA1c and 95% credible intervals

Triglyceride-rich lipoproteins, ranging from extremely large to very small VLDL particles, showed similar patterns of associations with HbA1c in prediabetic subjects with European, African Surinamese and Ghanaian background. In all three ethnic backgrounds, we observed that increasing concentrations of HbA1c were associated with increasing concentrations of large VLDL particles (Fig. 4).

In diabetic subjects, the association of HbA1c with triglyceride-rich lipoproteins was smaller compared to prediabetic subjects. We observed that increasing concentrations of extremely large VLDL remained associated with increasing of HbA1c in diabetic subjects with European background (males 0.47 [0.19–0.76], females 0.46 [0.16–0.74]) (Fig. 4).

##### Cholesterol-rich lipoproteins

**Fig. 5:**
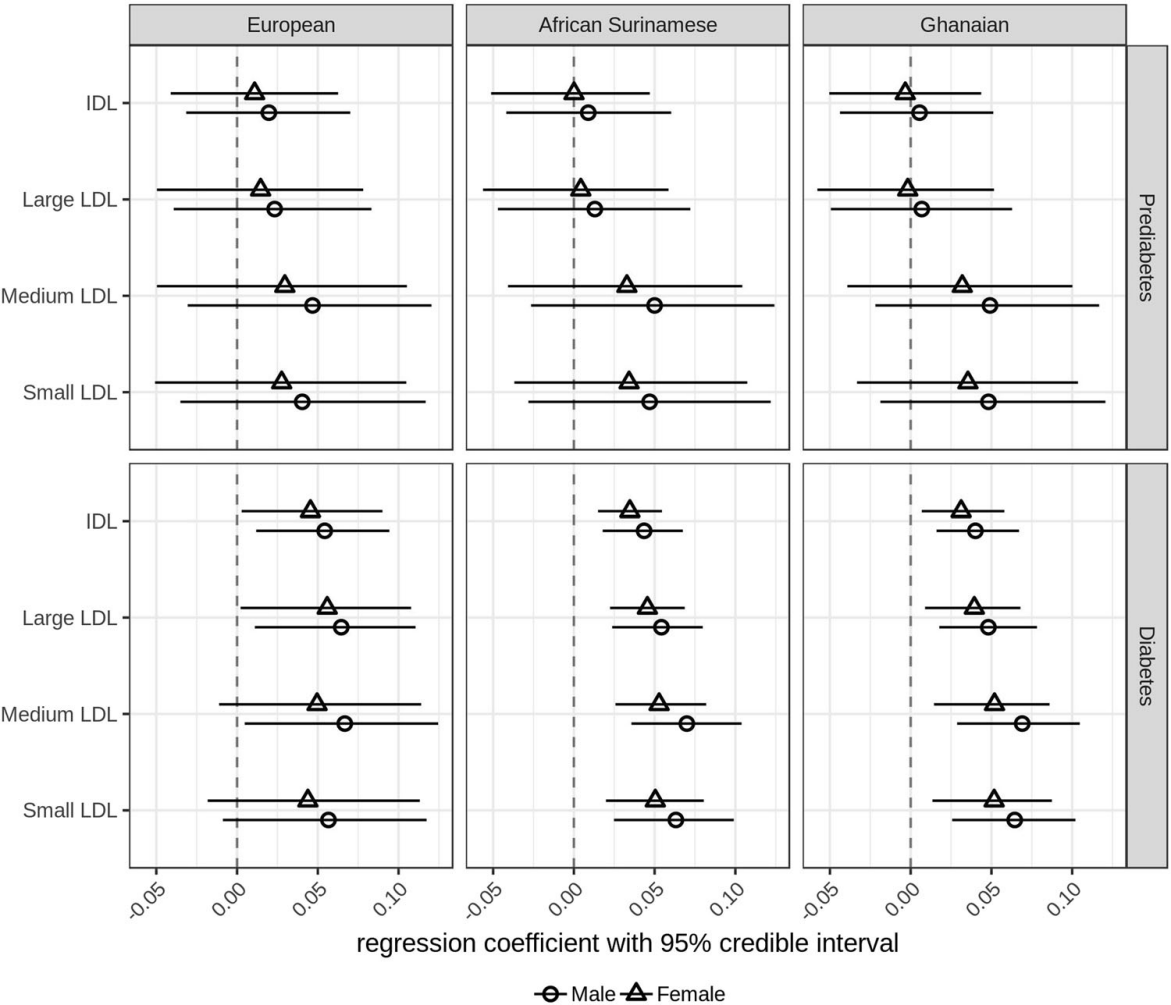
**Regression parameter estimates between plasma IDL and LDL particle concentration and HbA1c in subjects with European, Ghanaian and African Surinamese background**. Circles (female) or triangles (male) and horizontal lines represent the posterior means of the regression coefficient between plasma IDL and LDL particle concentration and HbA1c and 95% credible intervals

Cholesterol-rich lipoproteins including IDL, large, medium and small LDL particles were not associated with HbA1c in prediabetic subjects with European, African Surinamese and Ghanaian background. However, in diabetic, subjects HbA1c tended to positively correlate with concentrations of these cholesterol-rich lipoproteins. In particular, we noticed that increasing concentrations of HbA1c were associated with increasing concentrations of small LDL particles in diabetic subjects with African Surinamese (males 0.06 [0.02–0.10], females 0.05 [0.02–0.08]) and Ghanaian (males 0.06 [0.03–0.10], females 0.05 [0.01–0.09]) background, but were not associated with small LDL particle concentrations in diabetic subjects with European background (Fig. 5).

## Discussion

Why do subjects with African background have excess burden of T2DM? As an initial step to address this question, we performed metabolite profiling in plasma of 773 subjects from three ethnic groups in the Dutch population (European, Ghanaian and African Surinamese background), and assessed the associations of HbA1c with metabolic profiles.

We started our analyses with associations between HbA1c and circulating amino acids, because amino acids such as BCAAs and AAAs are robust biomarkers for prediabetes and diabetes^11^. Similar to other studies, we observed that increasing concentrations of HbA1c were associated with increasing concentrations of BCAAs (isoleucine, leucine and valine) as well as tyrosine in prediabetic participants with European background. Compared to prediabetic subjects with European background, we observed that associations between HbA1c and BCAAs were attenuated in diabetic subjects with European background. This attenuation might be attributable to medication or lifestyle changes.

Compared to participants with European background, associations of HbA1c with circulating amino acids were similar but less strong in subjects with African Surinamese and Ghanaian background. Interestingly, we observed that increasing concentrations of HbA1c were associated with decreasing concentrations of tyrosine in African Surinamese women with diabetes, but were not associated with this amino acid in diabetic participants with European background. It is not clear why the association of HbA1c and tyrosine in diabetic women with African Surinamese background was negative. Further research is needed to understand the role of tyrosine metabolism in hyperglycemia.

Overall, the less prominent associations between HbA1c and circulating amino acids in subjects with African background did not explain why they have excess burden of T2DM compared to subjects with European background. Therefore, we assumed that other metabolic abnormalities may underlie the excess burden of T2DM in subjects with African background.

In the next step, we evaluated associations of HbA1c with circulating ketone bodies (acetoacetate and 3-hydroxybutyrate). In our analysis, we observed that increasing concentrations of HbA1c were associated with decreasing concentrations of acetoacetate in diabetic subjects with European background, but were associated with increasing concentrations of acetoacetate in diabetic subjects with Ghanaian background, as well as in diabetic men with African Surinamese background. The effects of ketone bodies on glucose metabolism is dependent on the glucose level^31^. It was shown that elevation of circulating ketone bodies stimulated insulin secretion in men when high glucose was established^41^. This is in line with our observation in diabetic subjects with European background. On the other hand, ketone bodies such as acetoacetate may also inhibit insulin secretion by suppressing expression of ABCA1 (a key player in cellular cholesterol efflux) and promote accumulation of intracellular cholesterol^42,43^. We speculate that our observation of positive associations between HbA1c and circulating acetoacetate in African participants may be linked to the insufficient ABCA1-mediated cholesterol efflux capacity.

ABCA1-mediated cholesterol efflux exports intracellular cholesterol to HDL particles. Therefore, we focused on the associations of HbA1c levels and HDL particle concentrations. Our observation that increasing HbA1c correlated with decreasing large HDL particles in prediabetic and diabetic subjects with European background was consistent with the notion that low circulating HDL particle levels contribute to the pathophysiology of T2DM and HDL elevation is a potential treatment for hyperglycemia^44^. However, in prediabetic subjects with African Surinamese (men and women) and Ghanaian (women) background, we observed that increasing concentrations of HbA1c were associated with increasing concentrations of small HDL particles. Such relationship was also observed in diabetic females with African Surinamese background. Small HDL particles are the most efficient particle in ABCA1-mediated cholesterol efflux^45^. We hypothesized that subjects with African background might have impaired cholesterol efflux capacity to small HDL particles. In order to compensate this impairment, a larger number of small HDL particles was produced. Given that increasing HbA1c correlated with increasing acetoacetate in diabetic subjects with African background, we further hypothesized that the impaired ABCA1-mediated cholesterol efflux was induced by acetoacetate.

ABCA1-mediated cholesterol efflux and LDLr-mediated cholesterol uptake together determine overall accumulation of intracellular cholesterol. To address the question if LDLr-mediated cholesterol uptake plays a role in excess burden of T2DM in subjects with African background, we assessed the associations between HbA1c and apolipoprotein B containing lipoproteins. There are two types of apolipoprotein B containing lipoproteins, TG-rich and cholesterol-rich lipoproteins.

We first focused on the TG-rich lipoproteins that ranged from extremely large to very small VLDL particles. Like what we observed with the amino acids, associations of HbA1c with TG-rich lipoproteins were stronger in prediabetic subjects than in diabetic subjects, and were stronger in subjects with European background than in subjects with African backgrounds. Based on these observations, we ruled out the possibility that excess burden of T2DM in subjects with African background was due to excess supply of TG-rich lipoproteins.

Cholesterol-rich lipoproteins included IDL, large, medium and small LDL particles. We observed that increasing concentrations of HbA1c were associated with increasing concentrations of small LDL particles in diabetic subjects with African Surinamese and Ghanaian background, but were not associated with small LDL particles in diabetic subjects with European background. It was shown that excess uptake of cholesterol mediated by LDL receptor leads to cholesterol accumulation in islet and beta cell dysfunction^29^.

Overall, we observed that an increase in HbA1c was associated with more small HDL particles and more small LDL particles in subjects with African background, indicating impaired ABCA1-mediated cholesterol efflux capacity as well as excess LDLr-mediated cholesterol accumulation. These metabolic abnormalities might be linked to the excess burden of T2DM in subjects with African background. Future research is needed to test our hypothesis.

### Strength and limitation

A strength of this study is the use of Bayesian imputation to take care of missing observations in both dependent and independent variables. Dropping cases with missing data is the default setting used by many data analysis programs. However, this is almost never appropriate because the dropped cases can bias the results. The advantage of Bayesian imputation is that it does not generate one or a few imputed values but a whole posterior distribution for each missing observation.

One limitation of this study is that the three ethnic groups were from two different cohort studies, with different time of sampling and measurement. As a consequence, we cannot directly compare metabolite variables between ethnic groups but did run association analyses within each ethnic group. Another limitation is that we did not have information on medication use, diet and physical activity of participants. To partially account for the impact, we used BMI as an extra covariate in our statistical model.

In conclusion, subjects with Ghanaian and African Surinamese background showed different associations of HbA1c with circulating acetoacetate, small HDL particles and small LDL particles compared to Dutch with European background. These metabolic abnormalities suggested that the excess burden of T2DM in subjects with African background may be due to impaired cholesterol efflux capacity and abnormal cholesterol uptake.

## Supplementary information

Table S1

Table S2

Table S3

Table S4

## Data Availability

The metabolomics and clinical data of subjects with Ghanaian and African Surinamese background are available by submitting a proposal to the HELIUS Executive Board as outlined at http://www.heliusstudy.nl/en/researchers/ collaboration. The metabolomics and clinical data of subjects with European background are available by contacting human functional genomics project (www.humanfunctionalgenomics.org).

## Code availability

The model file and analysis code are available at https://github.com/XiangZhangSC/nutrition_and_diabetes_paper.

## References

[1] NCD Risk Factor Collaboration (NCD-RisC). (2016). Worldwide trends in diabetes since 1980: a pooled analysis of 751 population-based studies with 4.4 million participants. Lancet.

[2] Maskarinec G (2009). Diabetes prevalence and body mass index differ by ethnicity: the Multiethnic Cohort. Ethn. Dis..

[3] Bindraban NR (2008). Prevalence of diabetes mellitus and the performance of a risk score among Hindustani Surinamese, African Surinamese and ethnic Dutch: a cross-sectional population-based study. BMC Public Health.

[4] Ujcic-Voortman JK, Schram MT, Bruggen MA, der Verhoeff AP, Baan CA (2009). Diabetes prevalence and risk factors among ethnic minorities. Eur. J. Public Health.

[5] Meeks KAC (2015). Prevalence of type 2 diabetes and its association with measures of body composition among African residents in the Netherlands–The HELIUS study. Diabetes Res. Clin. Pract..

[6] Waters Kevin M., Stram Daniel O., Hassanein Mohamed T., Le Marchand Loïc, Wilkens Lynne R., Maskarinec Gertraud, Monroe Kristine R., Kolonel Laurence N., Altshuler David, Henderson Brian E., Haiman Christopher A. (2010). Consistent Association of Type 2 Diabetes Risk Variants Found in Europeans in Diverse Racial and Ethnic Groups. PLoS Genetics.

[7] Huisman MJ (2018). Does a high sugar high fat dietary pattern explain the unequal burden in prevalence of type 2 diabetes in a multi-ethnic population in The Netherlands? The HELIUS Study. Nutrients.

[8] Tillin T (2015). Diabetes risk and amino acid profiles: cross-sectional and prospective analyses of ethnicity, amino acids and diabetes in a South Asian and European cohort from the SABRE (Southall And Brent REvisited) Study. Diabetologia.

[9] Valkengoed van IGM (2017). Ethnic differences in metabolite signatures and type 2 diabetes: a nested case-control analysis among people of South Asian, African and European origin. Nutr. Diabetes.

[10] Roberts LD, Koulman A, Griffin JL (2014). Towards metabolic biomarkers of insulin resistance and type 2 diabetes: progress from the metabolome. Lancet Diabetes Endocrinol..

[11] Guasch-Ferré M (2016). Metabolomics in prediabetes and diabetes: a systematic review and meta-analysis. Diabetes Care.

[12] Wang TJ (2011). Metabolite profiles and the risk of developing diabetes. Nat. Med..

[13] Rebholz CM (2018). Serum metabolomic profile of incident diabetes. Diabetologia.

[14] Newgard CB (2009). A branched-chain amino acid-related metabolic signature that differentiates obese and lean humans and contributes to insulin resistance. Cell Metab..

[15] Newgard CB (2017). Metabolomics and metabolic diseases: where do we stand?. Cell Metab..

[16] Würtz P (2012). Metabolic signatures of insulin resistance in 7,098 young adults. Diabetes.

[17] Würtz P (2012). Circulating metabolite predictors of glycemia in middle-aged men and women. Diabetes Care.

[18] Würtz P (2013). Branched-chain and aromatic amino acids are predictors of insulin resistance in young adults. Diabetes Care.

[19] Lynch CJ, Adams SH (2014). Branched-chain amino acids in metabolic signalling and insulin resistance. Nat. Rev. Endocrinol..

[20] Wang TJ (2013). 2-Aminoadipic acid is a biomarker for diabetes risk. J. Clin. Invest..

[21] Fizelova M (2015). Associations of multiple lipoprotein and apolipoprotein measures with worsening of glycemia and incident type 2 diabetes in 6607 non-diabetic Finnish men. Atherosclerosis.

[22] Festa A (2005). Nuclear magnetic resonance lipoprotein abnormalities in prediabetic subjects in the Insulin Resistance Atherosclerosis Study. Circulation.

[23] Mackey RH (2015). Lipoprotein particles and incident type 2 diabetes in the multi-ethnic study of atherosclerosis. Diabetes Care.

[24] Wang J (2012). Lipoprotein subclass profiles in individuals with varying degrees of glucose tolerance: a population-based study of 9399 Finnish men. J. Intern. Med..

[25] Garvey WT (2003). Effects of insulin resistance and type 2 diabetes on lipoprotein subclass particle size and concentration determined by nuclear magnetic resonance. Diabetes.

[26] Eckardstein A, von Sibler RA (2011). Possible contributions of lipoproteins and cholesterol to the pathogenesis of diabetes mellitus type 2. Curr. Opin. Lipidol..

[27] Fryirs MA (2010). Effects of high-density lipoproteins on pancreatic beta-cell insulin secretion. Arterioscler. Thromb. Vasc. Biol..

[28] Kruit JK (2011). Islet cholesterol accumulation due to loss of ABCA1 leads to impaired exocytosis of insulin granules. Diabetes.

[29] Kruit JK (2010). Cholesterol efflux via ATP-binding cassette transporter A1 (ABCA1) and cholesterol uptake via the LDL receptor influences cholesterol-induced impairment of beta cell function in mice. Diabetologia.

[30] Stancáková A (2012). Hyperglycemia and a common variant of GCKR are associated with the levels of eight amino acids in 9,369 Finnish men. Diabetes.

[31] Mahendran Y (2013). Association of ketone body levels with hyperglycemia and type 2 diabetes in 9,398 Finnish men. Diabetes.

[32] Nathan DM, Singer DE, Hurxthal K, Goodson JD (1984). The clinical information value of the glycosylated hemoglobin assay. N. Engl. J. Med..

[33] Diabetes Prevention Program Research Group. (2015). HbA1c as a predictor of diabetes and as an outcome in the diabetes prevention program: a randomized clinical trial. Diabetes Care.

[34] Stronks K (2013). Unravelling the impact of ethnicity on health in Europe: the HELIUS study. BMC Public Health.

[35] Snijder MB (2017). Cohort profile: the healthy life in an urban setting (HELIUS) study in Amsterdam, The Netherlands. BMJ Open.

[36] Kurstjens S (2019). Increased NEFA levels reduce blood Mg2 + in hypertriacylglycerolaemic states via direct binding of NEFA to Mg2. Diabetologia.

[37] Netea MG (2016). Understanding human immune function using the resources from the Human Functional Genomics Project. Nat. Med..

[38] Inouye M (2010). Metabonomic, transcriptomic, and genomic variation of a population cohort. Mol. Syst. Biol..

[39] Dekker LH (2015). Comparable dietary patterns describe dietary behavior across ethnic groups in the netherlands, but different elements in the diet are associated with glycated hemoglobin and fasting glucose concentrations. J. Nutr..

[40] Carpenter B (2017). Stan: a probabilistic programming language. J. Stat. Softw. Artic..

[41] Jenkins DJ, Hunter WM, Goff DV (1970). Ketone bodies and evidence for increased insulin secretion. Nature.

[42] Uehara Y (2002). Polyunsaturated fatty acids and acetoacetate downregulate the expression of the ATP-binding cassette transporter A1. Diabetes.

[43] Brunham LR (2007). Beta-cell ABCA1 influences insulin secretion, glucose homeostasis and response to thiazolidinedione treatment. Nat. Med..

[44] Drew BG, Rye KA, Duffy SJ, Barter P, Kingwell BA (2012). The emerging role of HDL in glucose metabolism. Nat. Rev. Endocrinol..

[45] Du XM (2015). HDL particle size is a critical determinant of ABCA1-mediated macrophage cellular cholesterol export. Circ. Res..

